# Efficacy and Safety of Pericapsular Nerve Group Block (PENG) in Hip Surgery Under General Anaesthesia: A Systematic Literature Review and Meta-Analysis

**DOI:** 10.3390/jcm14020468

**Published:** 2025-01-13

**Authors:** Chryssoula Staikou, Martina Rekatsina, Matteo Luigi Giuseppe Leoni, Christos Chamos, Ioannis Kapsokalyvas, Giustino Varrassi, Iosifina Karmaniolou

**Affiliations:** 1 Department of Anesthesia, Aretaieio University Hospital, 11528 Athens, Greece; c_staikou@yahoo.gr; 2 Department of Medical and Surgical Sciences and Translational Medicine, Sapienza University of Rome, 00185 Rome, Italy; matteolg.leoni@gmail.com; 3 Department of Anaesthetics, Guy’s and St Thomas NHS Foundation Trust, London SE1 9RT, UK; christos.chamos@gstt.nhs.uk (C.C.); ioannis.kapsokalyvas@gstt.nhs.uk (I.K.); iosifina.karmaniolou@gstt.nhs.uk (I.K.); 4 Paolo Procacci Foundation, 00193 Rome, Italy; g.varrassi@fondazioneprocacci.org

**Keywords:** pericapsular nerve group block, PENG block, general anaesthesia, meta-analysis

## Abstract

**Background**: The pericapsular nerve group (PENG) block is a novel ultrasound-guided regional technique that may provide analgesia to patients undergoing hip surgery. It has been extensively studied in recent years, but the evidence of superiority over other regional anaesthetic techniques is inconclusive. This review aimed to compare outcomes of the PENG block in patients undergoing hip surgery with standard techniques under general anaesthesia. **Methods**: PRISMA (Preferred Reporting Items for Systematic Reviews and Meta-Analyses) guidelines were followed throughout the preparation of this review. Randomised trials from electronic databases were included. We investigated postoperative pain scores, required analgesia, and adverse events associated with the block. **Results**: Ten studies satisfied the criteria to be included in the meta-analysis. Data from 646 patients were analysed, in which 321 patients received PENG block and 325 were included in the comparative groups. Pain scores at rest, at 24 h (*p* = 0.04) and 48 h (*p* = 0.02) were lower in patients who had received the PENG block. This group also required a smaller amount of opioids at 24 h after the procedure, but this difference was not statistically significant (*p* = 0.53); while a similar non-significant reduction in opioid consumption was also observed at 48 h. Although PENG seems to delay the time to the first analgesic request, we failed to prove a statistically significant difference (*p* = 0.83). Patient satisfaction also seems to be better in the PENG group, but not in a statistically significant way. No important side effects related to the block were described. **Conclusions**: PENG block for major hip surgery offers better postoperative analgesia, with possibly less opioid consumption. It seems to prolong the time to the first analgesic but does not significantly affect common side effects of anaesthesia/analgesia such as PONV or duration of hospital stay.

## 1. Introduction

Hip surgery, including hip arthroplasty and hip fracture surgery, is a common procedure with a high success rate, mainly performed in older adults and offers significant benefits to patients such as pain relief, increased active and passive mobility and ability to walk [1].

Nevertheless, hip surgery may be accompanied by postoperative pain that restricts early ambulation and increases the risk for adverse events (i.e., thromboembolism, cardio-pulmonary complications), which prolongs hospital stay [1,2]. Opioid analgesia, despite being the mainstay of pain management after surgery, may incur multiple unwanted effects such as sedation, dizziness, lethargy, respiratory depression, or gastrointestinal complications [3].

Central neuraxial blocks [4], local analgesia (i.e., surgical site infiltration) [5,6] and a variety of peripheral nerve blocks have been proposed to offer better quality of analgesia and decrease opioid consumption [7,8,9]. Despite all the different available modalities, the optimal analgesic strategy remains undetermined [8]. The complexity of hip joint innervation [10], along with side effects of current regional techniques, such as quadriceps muscle weakness and consequent falls [11], require the development of new alternative methods.

The pericapsular nerve group (PENG) block is a novel, ultrasound-guided, regional technique that may provide effective and safe analgesia to patients undergoing hip surgery [12]. It targets the articular branches of the femoral, obturator and accessory obturator nerve that provide sensory innervation to the anterior hip capsule, offering high-quality analgesia without motor block [12]. Many investigators have highlighted the abovementioned benefits of the PENG block for different types of hip surgery [13,14]. Nevertheless, cases of motor weakness have also been reported [15,16].

Overall, the evidence has been inconclusive. This systematic review and meta-analysis aim to clarify the efficacy and safety of the PENG block in patients undergoing hip surgery under general anaesthesia.

## 2. Materials and Methods

### 2.1. Search Strategy

We adopted the Preferred Reporting Items for Systematic Reviews and Meta-Analyses (PRISMA) recommendations for the preparation of this review [17]. Randomised trials that investigated the efficiency of the PENG block for hip surgery under general anaesthesia were reviewed and evaluated using a predesigned protocol. The protocol was registered with the International Prospective Register of Systematic Reviews (PROSPERO-CRD42022335593).

Two authors (I.K. and M.R.) separately performed an exhaustive search for articles published from January 1990 until July 2024 in the electronic databases MEDLINE, Embase, PubMed, CINAHL, Cochrane Database of Systematic Reviews and clinicaltrials.gov. The search terms “pericapsular nerve block” or “PENG block” in combination with “hip surgery”, “hip fracture surgery”, “hip replacement”, “hip prosthesis”, “hip operation”, “hip arthroplasty”, “general anaesthesia” in all possible combinations, were used. The searches were re-run just before the final analyses and any further studies were identified. Additionally, manual searching for relevant publications of RTCs was conducted. The same two authors evaluated separately the abstracts retrieved; when agreement was not reached the opinion of a third author (C.S.) was obtained.

### 2.2. Eligibility Criteria

We included randomised trials studying the impact of PENG block for hip surgery (total hip arthroplasty and hip fracture) conducted under general anaesthesia in adult patients over 18 years old, published from January 1990 until July 2024. Only trials published in English, which clearly mentioned approval from the local ethics committee or the institutional review board, were considered eligible.

### 2.3. Data Extraction

A standardised data collection form was produced using Microsoft Excel 2016 (Microsoft Corp, Redmond, WA, USA). It was used for data extraction and entry by 3 authors (C.H., I.K., M.R.). Discrepancies in data extraction were resolved by a fourth author (C.S.). Data were sourced primarily from published tables or the manuscript text. If required data were missing, we attempted to contact the authors for clarification or extract from published figures using online plot digitiser software (WebPlotDigitizer 3.9, Ankit Rohatgi, 2015, Austin, TX, USA).

### 2.4. Outcomes Assessed

Our primary outcome was postoperative pain at 6, 24, and 48 h after surgery. Postoperative pain was assessed using both the resting pain score (static) and the pain score during movement (dynamic). Secondary outcomes were postoperative cumulative opioid consumption at 24 and 48 h after surgery, time to first analgesia request, duration of surgery and the occurrence of postoperative nausea and vomiting.

### 2.5. Risk of Bias Assessment in Included Studies

All studies were initially screened for bias using the JADAD scoring system. Trials with Jadad score < 4 were excluded. Studies with a Jadad score ≥ 4 were further assessed using the Cochrane Collaboration risk of bias assessment tool Version 2 (RoB2) [18]. The studies were independently assessed by I.K. and M.R. A score was assigned to each study by consensus; if an agreement could not be reached, the third author (C.S.) was again consulted. The risk of publication bias was assessed by visual examination of the funnel plot as well as by the Egger test [19].

### 2.6. Statistical Analysis

This meta-analysis was performed in line with recommendations from the Cochrane Collaboration and the PRISMA Statement [20]. The random effects model was used due to the different sets of studies (different control groups). As the overall effect estimate for continuous variables, we used the standardised mean difference (SMD) with 95% confidence intervals (CI). This approach was applied when the same outcome was measured using different units across studies when there were variations in the distribution of continuous variables, or when the possibility of non-normal data distribution across studies could not be ruled out. Mean difference with 95% confidence intervals was used to provide a direct comparison of outcomes measured on the same scale across studies, ensuring that the results reflect the absolute difference between groups. As an overall effect estimate of binary variables, we used the odds ratio. The heterogeneity was assessed using the I^2^, the tau^2^ and Cochran’s Q-test along with the degrees of freedom and the corresponding *p*-value. An I^2^ value higher than 50% or a statistically significant Cochran’s Q-test were indicative of substantial heterogeneity [21]. Possible sources of heterogeneity within the outcome were identified in advance and included as covariates in the planned meta-regression analysis. The included covariates were skin infiltration for local analgesia, intraoperative opioid use and dose of local anaesthetic use for PENG block. For continuous variables reported as medians with interquartile ranges, these data were transformed into means and standard deviations following the guidelines provided by Wan et al. [22], as well as the Cochrane Handbook for systematic reviews of interventions [21]. Postoperative opioid use was converted to oral morphine equivalents (OME) to standardise the measurement across different types of opioids. Patient satisfaction scores not originally reported on a 10-point scale were converted to continuous values using linear transformation [23]. R software v4.3.2 (R Foundation for Statistical Computing, Vienna, Austria, www.r-project.org) was used for the analyses, and the level of statistical significance was set to 0.05.

## 3. Results

### 3.1. Study Characteristics

Of the 744 studies identified, 10 satisfied the inclusion criteria (Supplementary Figure S1). Data from 646 patients were analysed, in which 321 patients received PENG block and 325 were included in the comparative groups. A description of the included studies is summarised in Table 1.

### 3.2. Risk of Bias

**Figure 1:**
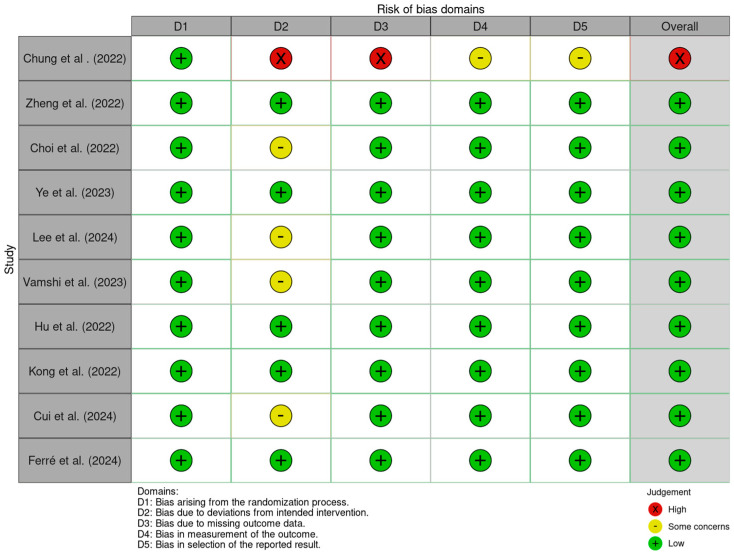
Summary of the risk of bias assessment for included studies across five domains using the Cochrane Risk of Bias 2.0 (RoB 2) tool [24,25,26,27,28,29,30,31,32,33]. Domains assessed include: D1 (bias arising from the randomisation process), D2 (bias due to deviations from the intended intervention), D3 (bias due to missing outcome data), D4 (bias in measurement of the outcome), and D5 (bias in the selection of the reported result). Judgments are categorised as low risk (green), some concerns (yellow), and high risk (red). The overall risk of bias for each study is also reported in the rightmost column.

The risk of bias table from the included studies is reported in Figure 1. Generally, included studies had an overall low risk of bias.

### 3.3. Primary Outcomes

As previously reported, postoperative static and dynamic pain scores at 6, 24, and 48 h after surgery were considered as primary outcomes.

### 3.4. Pain Score at Rest (Static)

**Figure 2:**
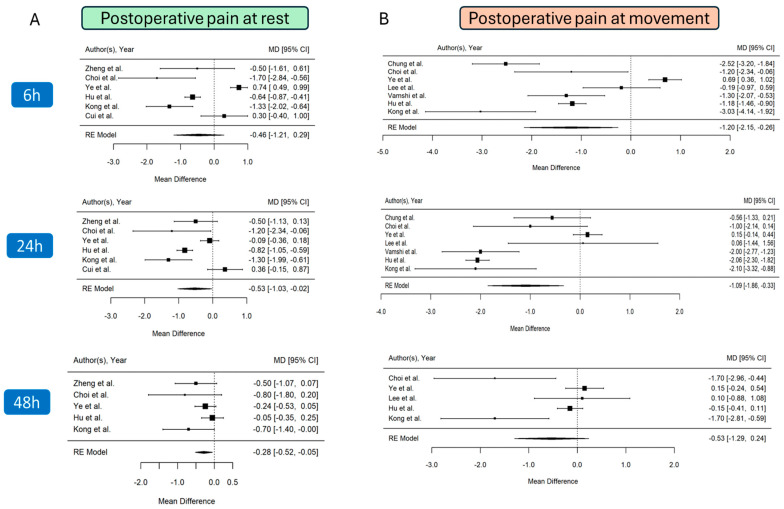
Forest plot displaying pooled effect sizes for postoperative VAS scores at rest (**A**) and dynamic pain scores (**B**), measured at 6, 24, and 48 h after surgery [24,25,26,27,28,29,30,31,32,33].

The cumulative pain scores at rest at 6, 24, and 48 h post-surgery were examined in five studies [24,25,26,27,28,29] (*n* = 416) for the 6 and 24 h time points, while four studies [24,25,26,27,28] (*n* = 344) reported data on pain intensity at 48 h post-surgery. Globally, the analysis indicated lower pain intensity in patients treated with the PENG block (Figure 2A). At 6 h, there was a non-significant reduction in pain of 0.46 on a 0–10 pain scale (MD, −0.46; 95% CI, −1.21 to 0.30; *p* = 0.23, I^2^ = 93%). However, at 24 h, the pain reduction became statistically significant, with a decrease of 0.53 (MD, −0.53; 95% CI, −1.03 to −0.02; *p* = 0.04, I^2^ = 87%). The pain scores during rest at 6 and 24 h after surgery were characterised by substantial heterogeneity. Sensitivity analysis showed that when the study of Ye et al. [26] was removed, the pooled results at 6 h follow-up were improved (MD, −0.72; 95% CI, −1.37 to −0.07; *p* = 0.03) while the heterogeneity was only slightly reduced (I^2^ = 78.6%), (Supplementary Figure S2). Meta-regression analysis for pain intensity at 6 h revealed no role for skin infiltration for local analgesia (*p* = 0.85), for the dose of local anaesthetic used for the block (*p* = 0.67), while intraoperative opioid use was statistically significant (*p* = 0.04).

Similar findings were observed at the 24 h follow-up, where the sensitivity analysis confirmed the significant influence of the study by Ye et al. [26] on the pooled results (MD, −0.64; 95% CI, −1.23 to −0.05; *p* = 0.03), (Supplementary Figure S2). However, heterogeneity was not significantly reduced (I² = 83%). The meta-regression analysis revealed no significant effect of skin infiltration for local analgesia (*p* = 0.70), the dose of local anaesthetic used for the block (*p* = 0.99), or intraoperative opioid use (*p* = 0.42). The VAS at 24 h follow-up should be interpreted cautiously, as the random effects model, sensitivity analysis, and meta-regression did not account for the variability in the effect size of the PENG block at 24 h post-surgery. Therefore, this outcome can be considered unstable. By 48 h, the pain reduction remained significant, though smaller, at 0.28 (MD, −0.28; 95% CI, −0.52 to −0.05; *p* = 0.02), with no evidence of heterogeneity (I^2^ = 25%).

No publication bias was observed through visual inspection of funnel plots and confirmed by Egger’s regression tests (6 h follow-up, Egger’s regression test: *p* = 0.21; 24 h follow-up, Egger’s regression test: *p* = 0.34; 48 h follow-up, Egger’s regression test: *p* = 0.23).

### 3.5. Pain Score at Movement (Dynamic)

The post-surgical dynamic pain score at 6 and 24 h was reported by seven studies [26,27,28,29,30,31,32] with a total of 444 patients. Data on pain intensity during movement at 48 h post-surgery were provided by five studies [25,26,27,28,31] involving 334 patients. Overall pain scores showed significant improvement at both 6 and 24 h after surgery. The reduction in pain intensity at 6 h was 1.20 (MD, −1.20; 95% CI, −2.15 to −0.26; *p* = 0.013, I^2^ = 95%), and at 24 h, it was 1.09 (MD, −1.09; 95% CI, −1.86 to −0.33; *p* = 0.005, I^2^ = 92%). However, no significant improvement was observed at the 48 h follow-up (MD, -0.53; 95% CI, −1.29 to 0.24; *p* = 0.18, I^2^ = 88%), as shown in Figure 2B. Similar to the pain scores at rest, the dynamic pain scores were affected by high heterogeneity. Sensitivity analysis did not alter the overall estimates of the dynamic pain score, indicating that the effect size was consistent across all studies, even though the overall heterogeneity remained high. Meta-regression analysis revealed a significant impact of intraoperative opioid use on pain intensity at both 6 h (*p* < 0.001) and 24 h (*p* = 0.003) post-surgery. However, no significant effects were found for skin infiltration for local analgesia or the dose of local anaesthetic used for the block.

### 3.6. Secondary Outcomes

Postoperative cumulative opioid consumption at 24 and 48 h after surgery

**Figure 3:**
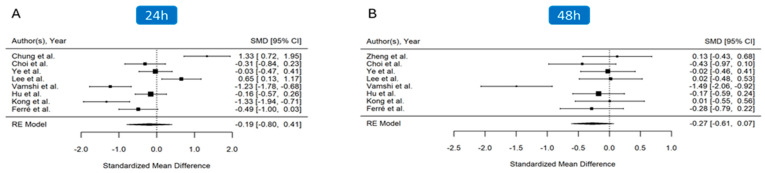
Forest plot displaying pooled effect sizes for postoperative cumulative opioid consumption at 24 and 48 h after surgery [24,25,26,27,28,30,31,32,33]. (**A**) At 24 h post-surgery, the PENG block group showed a reduction in cumulative opioid consumption, though this reduction did not reach statistical significance. (**B**) By 48 h, the reduction in opioid consumption remained non-significant. Substantial heterogeneity was present among studies.

The amount of opioid analgesia required after surgery at 24 h was reported by eight studies [25,26,27,28,30,31,32,33] (n = 504), while a similar set of eight studies reported the 48 h opioid consumption [24,25,26,27,28,31,32,33] (n = 524). Overall, the analysis indicated lower cumulative opioid consumption at 24 h in patients treated with the PENG block even if not statistically significant (SMD, -0.19; 95% CI, -0.80 to 0.41; *p* = 0.53, I^2^ = 91%), (Figure 3A). Sensitivity analysis did not change the overall estimates of the cumulative opioid consumption at 24 h, indicating that the effect size is similar across all studies. Meta-regression analysis revealed a significant role in intraoperative opioid use (*p* = 0.002), while no significant effect was observed for skin infiltration for local analgesia (*p* = 0.64) and the dose of local anaesthetic used for the block (*p* = 0.47).

At 48 h, the same non-significant reduction in opioid consumption was observed (SMD, −0.27; 95% CI, −0.61 to 0.07; *p* = 0.12, I^2^ = 73%) (Figure 3B). The sensitivity analysis did not alter the overall estimates of cumulative opioid consumption at 48 h. Meta-regression analysis demonstrated a non-significant effect for the following variables: intraoperative opioid use (*p* = 0.14), skin infiltration for local analgesia (*p* = 0.59), and the dose of local anaesthetic used for the block (*p* = 0.36).

### 3.7. Time to First Analgesia Request

**Figure 4:**
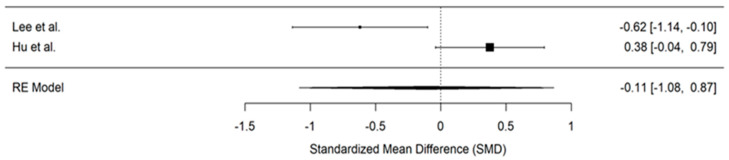
Forest plot displaying pooled effect sizes for time to first analgesia request post-surgery [27,31].

Time to first analgesia request was reported by only two studies [27,31], while Chung et al. [30] only provided data on the incidence of analgesic requests within a specific time range, which did not allow for effect size calculation. As a result, pooling the results from the two studies [27,31] showed a reduction in the time to the first analgesic request in the PENG group compared to the control group. However, this reduction was not statistically significant (SMD, −0.11; 95% CI, −1.08 to 0.87; *p* = 0.83, I^2^ = 88%), (Figure 4). Given that only two studies were included, neither sensitivity analysis nor meta-regression was possible.

### 3.8. Duration of Surgery

**Figure 5:**
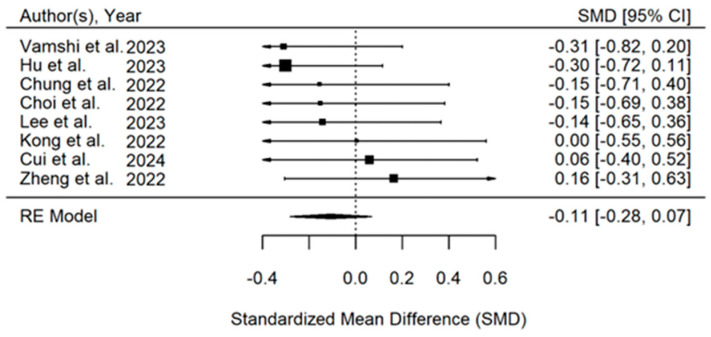
Forest plot displaying pooled effect sizes for the duration of surgery in patients receiving PENG block versus control. The analysis included eight studies [24,25,27,28,29,30,31,32] and showed no significant difference in the duration of surgery between the PENG block group and controls.

The duration of surgery was reported by eight articles [24,25,27,28,29,30,31,32] and averaged 88 ± 20 min. No significant difference in the duration of surgery was observed in the overall analysis between patients who received the PENG block and controls (SMD, −0.11; 95% CI, −0.28 to 0.07; *p* = 0.23, I^2^ = 0%), (Figure 5).

### 3.9. Patient Satisfaction

**Figure 6:**
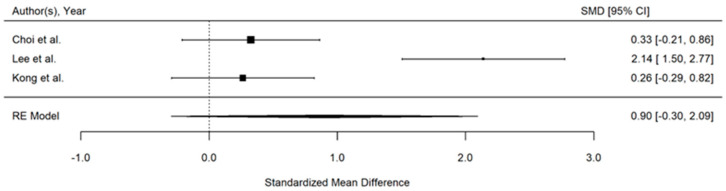
Forest plot displaying the pooled effect sizes for patient satisfaction [25,28,31]. The satisfaction data were standardised to a common scale (0 to 10) for comparison. Although there was an increase in satisfaction levels among patients who received the PENG block, this improvement did not reach statistical significance.

Patient satisfaction was assessed in three studies [25,28,31], though using different rating scales. After standardising the data to a common scale (0 to 10), the overall effect size indicated higher satisfaction levels among patients who received the PENG block. However, this increase in satisfaction did not reach statistical significance (SMD, 0.90; 95% CI, −0.30 to 2.09; *p* = 0.14), and there was considerable heterogeneity among the studies (I^2^ = 92%), (Figure 6).

### 3.10. Length of Hospital Stay

**Figure 7:**
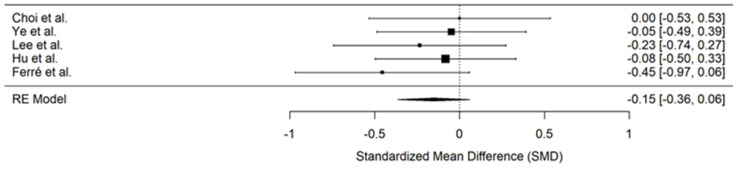
Forest plot displaying pooled effect sizes for the length of hospital stay (in hours) [25,26,27,31,33]. The PENG block group showed a slight reduction in the length of hospital stay compared to the control group, but this reduction was not statistically significant.

Five studies reported the length of hospital stay [25,26,27,31,33]. To enable a direct comparison between studies, the length of stay was standardised to hours across all reports. All patients demonstrated a similar length of hospital stay, and the PENG block treatment did not significantly affect this outcome (SMD −0.15; 95% CI, −0.36 to 0.06, *p* = 0.16, I^2^ = 0%) (Figure 7). There was no observed heterogeneity among the studies.

### 3.11. Occurrence of Postoperative Nausea and Vomiting

**Figure 8:**
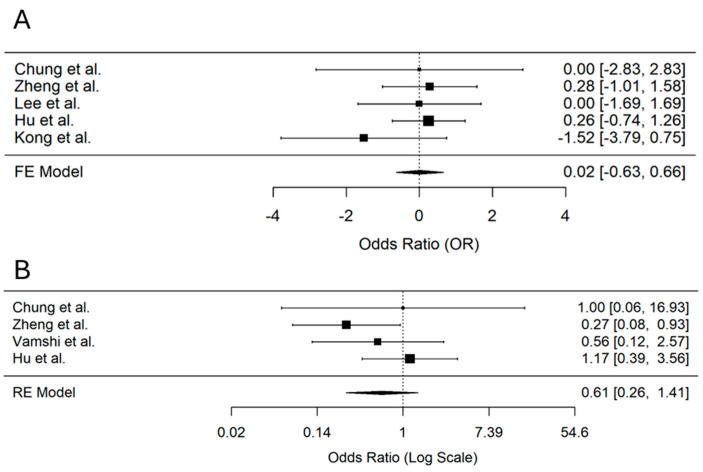
Forest plots displaying the pooled odds ratios (OR) for postoperative nausea (**A**) and vomiting (**B**) in patients receiving PENG block versus control [24,27,28,30,31,32].

Occurrence of postoperative nausea was reported in five studies [24,27,28,30,31]. No significant reduction in the odds of nausea was observed in the PENG block group compared to controls (OR 0.02, 95% CI −0.63 to 0.66; *p* = 0.96, I² = 0%), (Figure 8A). Similarly, four studies [24,27,30,32] reported the occurrence of vomiting, and no significant reduction was observed in the PENG block group (OR 0.61, 95% CI 0.26 to 1.41; *p* = 0.25, I² = 23.3%), (Figure 8B).

## 4. Discussion

The PENG block has been favoured in hip surgery for its analgesic efficacy and purported motor-sparing properties [34]. This meta-analysis included data from 10 randomised trials regarding the efficacy and safety of this recently introduced block for patients undergoing hip surgery. According to our results, PENG block is beneficial in terms of reducing postoperative pain intensity at 24 and 48 h. Specifically, pain at rest was significantly reduced at 24 and 48 h after surgery in patients treated with the PENG block, with significant heterogeneity at earlier time points but reduced variability at 48 h. Dynamic pain scores showed significant improvement at both 6 and 24 h post-surgery; however, this benefit was not sustained at 48 h, with high heterogeneity persisting across all time points.

Opioid consumption was one of the secondary outcomes of our study; the meta-analysis did not show a statistically significant difference among groups, even though the overall consumption of opioids was higher in the non-PENG block groups, both at 24 and at 48 h. PENG block was not associated with a significant prolongation of time to the first analgesic request postoperatively. However, the meta-regression analysis identified a significant role of intraoperative opioid use in influencing several outcomes: static pain scores at 6 h, dynamic pain scores at 6 and 24 h post-surgery, as well as postoperative cumulative opioid consumption at 24 and 48 h after surgery. The incidence of postoperative nausea and vomiting was similar between the groups. Similarly, the length of hospital stay was not affected. Patient satisfaction was only mentioned in three studies. Higher satisfaction was reported by patients who received a PENG block; however, this did not reach statistical significance.

As a novel modality, PENG block has been compared to other locoregional analgesic techniques, such as lumbar plexus block [31], fascia iliaca [13,14,25,29,32], or other mini-invasive analgesic methods (i.e., periarticular infiltration) [26,33]. Importantly, this block has been described to be performed under ultrasound guidance [12] so that the iliopubic eminence, the iliopsoas muscle and tendon, the femoral artery, and the pectineus muscle are observed. The local anaesthetic is administered in the musculofascial plane between the psoas tendon anteriorly and the pubic ramus posteriorly in increments under ultrasound vision [12]. In the future, technological advancements such as three-dimensional reconstruction imaging and magnetic resonance microscopy of peripheral nerves may play a significant role in peripheral nerve blocks and plane blocks [35].

The results of this meta-analysis showed that compared to other blocks or conventional opioid-based analgesia, PENG block was associated with better postoperative analgesia at 24 and 48 h. At 6 h after the surgical procedure pain at rest did not show a significant difference, but pain at movement was significantly reduced. The abovementioned findings on pain intensity differ from those reported by Huda et al. [36] in their meta-analysis. The authors found that PENG block offered no significant pain relief compared to other methods up to 24 h postoperatively. Since we have no results regarding pain during the immediate postoperative period, we cannot compare our findings to those of Farag et al. [37], who reported in their meta-analysis that at 30 min postoperatively dynamic pain was significantly lower using PENG block compared to other blocks or parenteral analgesia. Interestingly, these authors found that the analgesic superiority of the block did not last and faded quickly; there was no difference in analgesia compared to the other methods at 6 h postoperatively or later. This result is also different from the findings of our research. The differences may be at least partly attributed to the different characteristics of the studies included in the two meta-analyses, such as type of surgery or setting (i.e., arthroscopy, scheduled or emergency cases, use of PENG in the emergency department), type of anaesthesia, and comparison of PENG block with different modalities (i.e., lumbar plexus block and other locoregional techniques) [13,14,25,26,29,31,32,33]. Still, despite the above discrepancies, both meta-analyses agree with our results that PENG block is associated with reduced opioid consumption in the first 24 postoperative hours [36,37]. Moreover, we found that this beneficial effect may last up to 48 h. Our results showed that even at 48 h, the analgesic consumption was less in patients who had a PENG block, even though the difference was not significant (*p* = 0.12).

In contrast to Huda et al. [36], we did not find that PENG block prolongs intraoperative analgesia, i.e., the time of request of the first analgesic dose after the procedure did not differ among the groups. This is in accordance with our finding that the PENG block did not reduce the cumulative opioid consumption 48 h after the procedure. However, this finding does not undermine the importance of better analgesia in the PENG group, especially when considering the growing evidence of postoperative pain chronification in patients with poor acute postoperative pain management [38,39].

Similar to previous results, we found no significant statistical difference in PONV [36]. Farag et al. [37], on the other hand, found no significant difference in nausea, pruritus, and dizziness, while patients who received a PENG block had significantly less vomiting. The small number of studies that reported this side effect might have prevented us from finding a difference between the groups.

Regarding hospital stay, the results of this meta-analysis are in accordance with previous findings, suggesting no effect of PENG block on time to patient discharge from hospital [36]. Also, we had no clear result on the possible superiority of the PENG block with regards to patient satisfaction, although according to previous meta-analyses, the PENG block was associated with higher scores of patient satisfaction [36,37].

### Limitations

This meta-analysis has a few limitations, mainly due to the small sample sizes of most studies and the different research protocols. The PENG block was compared to other blocks or analgesic methods. The included studies differed in their methodology regarding the comparator groups since a few used active comparator groups (i.e., different blocks), while others used non-block control groups or sham control groups. Also, the studies differed in the local anaesthetic used as well as the doses/volumes and concentrations of the injected solution.

Moreover, the type of surgery (total hip arthroplasty and hip fracture surgery), as well as the surgical teams, were not the same in all studies. Nevertheless, we consider that the severity of the procedures was similar since only studies with hip fracture surgery and total hip arthroplasty were included in the analysis. The heterogeneity of studies is a common limitation in most meta-analyses. To minimise data variability, we applied strict inclusion criteria in our search strategy and study evaluation process. Additionally, meta-regression analyses were conducted to further investigate the potential sources of heterogeneity. Finally, we had no adequate data to analyse analgesia in the immediate and early postoperative period.

## 5. Conclusions

In conclusion, according to our meta-analysis, the PENG block used in major hip surgery offers better postoperative analgesia; however, less opioid consumption is not warranted. It does not prolong the time to the first analgesic, and it does not seem to affect significantly common side effects of anaesthesia/analgesia such as PONV, or the duration of hospital stay. As a novel technique, PENG block can be described as a safe and effective regional block technique for patients undergoing hip fracture surgery. However, the existing evidence is not adequate to draw firm conclusions. More high-quality data from large, well-designed randomised clinical trials, as well as cadaveric and anatomical trials, will be needed and further analyses are required to confirm the superiority of the PENG block over other locoregional techniques or conventional analgesic modalities used in hip surgery.

## Figures and Tables

**Table 1:** Studies’ characteristics.

Study (Year)	Patients (PENG/ Control)	Type of Surgery	Type of Anaesthesia	PENG group: PENG only or PENG + LIA	Control Group	Local Anaesthetic Used (Concentration)	Dose of local Anaesthetic for the PENG Block	Adjuvants in PENG	Local Infiltration Analgesia Regimen	Intraoperative Opioid	Postoperative Opioids
Zheng et al. (2022) [24]	34/36	THA	GA	PENG + LIA	PENG saline	Ropivacaine 0.5%	100 mg		all patients: intra-articular infiltration 20 mL of 0.5% ropivacaine	Y (sufentanil 0.5–0.7 μg/kg)	Sufentanil (2.5 mcg increments), intravenous tramadol/butorphanol/meperidine allowed
Choi et al. (2022) [25]	27/27	THA	GA	PENG	Fascia iliaca compartment block	Ropivacaine 0.2%	40 mg	adrenaline 1:200,000	Not given	Y (fentanyl 1 mcg/kgl and remifentanil)	PCA Fentanyl
Ye et al., 2023 [26]	40/40	THA	GA	PENG	LIA	Ropivacaine 0.5%	100 mg	adrenaline 1:200,000	only control group infiltration of joint capsule with 0.33% ropivacaine, 1:200,000 adrenaline, total 30 mL	GA regimen not mentioned	Morphine PRN
Hu et al., 2023 [27]	45/45	THA	GA	PENG+LIA	Sham PENG block + LIA	Ropivacaine 0.2%	40 mg	adrenaline 1:200,000	LIA to both groups (patients in the PENG group received 20 mL of 0.5% ropivacaine with 1:200,000 epinephrine, while those in the Sham group received 40 mL.)	Y (sufentanil 0.3 mg/kg and remifentanil)	Morphine SC (rescue)
Kong et al., 2022 [28]	25/25	THA	GA	PENG+LIA	Fascia iliaca compartment block	Ropivacaine 0.375%	112.5 mg		all patients received 15 mL of 0.375% ropivacaine for local infiltration anaesthesia before suturing	Y (fentanyl 1 mcg/kg and remifentanil)	PCA Fentanyl
Cui et al., 2024 [29]	36/36	Hip arthroplasty	GA	PENG	Supra-inguinal fascia iliaca	Ropivacaine 0.3%	60 mg		Not mentioned	Y (sufentanil 0.4 mcg/kg + extra sufentanil 0.05 mcg/kg if needed + sufentanil 0.1 mcg/kg before end of surgery and remifentanil)	PCA Sufentanil
Chung et al. (2022) [30]	25/25	THA	GA	PENG+LIA	PENG saline	Ropivacaine 0.5%	125 mg		All patients received wound infiltration of 20mL ropivacaine 0.375% from the surgeon at the end of the surgical procedure	Y (fentanyl 0.9 mcg/kg))	PCA Fentanyl
Lee et al., 2023 [31]	30/30	Hip fracture	GA	PENG+LIA	Lumbar Plexus Block + LIA	Ropivacaine 0.5%	100 mg		All patients (wound infiltration and a periarticular injection of 20 mL of 0.25% ropivacaine with ketorolac (30 mg) at the end of the surgical procedure)	GA regimen not mentioned	PCA Fentanyl
Vamshi et al., 2023 [32]	30/30	THA	GA	PENG	Suprainguinal fascia iliaca block	Bupivacaine 0.25%	75 mg	1 mcg/kg clonidine diluted in bupivacaine solution	Not mentioned	Y (fentanyl 2 microgram/kg + additional bolus of 0.5 mcg/kg as needed)	PCA Morphine
Ferré et al., 2024 [33]	29/31	THA	GA	PENG+LIA	LIA	Ropivacaine 0.475%	95 mg		All patients received 80 mL of ropivacaine 2 mg/ mL (for a total dose of 160 mg	Y (sufentanil if necessary 2.5–7.5 mcg)	Oxycodone PRN

PENG = pericapsular nerve group, LIA = local infiltration analgesia, THA = total hip arthroplasty, GA = general anaesthesia, PCA = Patient Control Analgesia, SC = subcutaneously, Y = YES, N = NO, PRN= pro re nata (as needed).

## Data Availability

Data are available upon request to the corresponding author.

## Supplementary Materials

The following supporting information can be downloaded at: https://www.mdpi.com/article/10.3390/jcm14020468/s1, Figure S1. PRISMA 2020 flow diagram for new systematic reviews which included searches of databases, registers and other sources; Figure S2. Sensitivity analysis of mean differences (MD) in pain scores during rest at 6 hours (A) and 24 hours (B) post-surgery.
