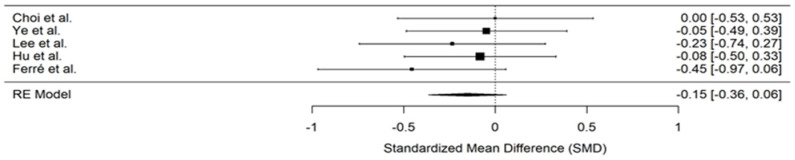# Correction: Staikou et al. Efficacy and Safety of Pericapsular Nerve Group Block (PENG) in Hip Surgery Under General Anaesthesia: A Systematic Literature Review and Meta-Analysis. *J. Clin. Med.* 2025, *14*, 468

**DOI:** 10.3390/jcm14061792

**Published:** 2025-03-07

**Authors:** Chryssoula Staikou, Martina Rekatsina, Matteo Luigi Giuseppe Leoni, Christos Chamos, Ioannis Kapsokalyvas, Giustino Varrassi, Iosifina Karmaniolou

**Affiliations:** 1 Department of Anesthesia, Aretaieio University Hospital, 11528 Athens, Greece; c_staikou@yahoo.gr; 2 Department of Medical and Surgical Sciences and Translational Medicine, Sapienza University of Rome, 00185 Rome, Italy; matteolg.leoni@gmail.com; 3 Department of Anaesthetics, Guy’s and St Thomas NHS Foundation Trust, London SE1 9RT, UK; christos.chamos@gstt.nhs.uk (C.C.); ioannis.kapsokalyvas@gstt.nhs.uk (I.K.); iosifina.karmaniolou@gstt.nhs.uk (I.K.); 4 Paolo Procacci Foundation, 00193 Rome, Italy; g.varrassi@fondazioneprocacci.org

**Error in Figure**

In the original publication, due to an editorial error, there is a mistake in Figure 7 [1]. Figures 6 and 7 were duplicated. The corrected Figure 7 appears below. The editorial office and the authors state that the scientific conclusions are unaffected. This correction was approved by the Academic Editor. The original publication has also been updated.