# Supplementary material for: Efficacy and Safety of Pericapsular Nerve Group Block (PENG) in Hip Surgery Under General Anaesthesia: A Systematic Literature Review and Meta-Analysis
Source: J Clin Med. 2025 Jan 13;14(2):468. doi: 10.3390/jcm14020468 (PMC11765790; doi:10.3390/jcm14020468)
Supplement: Supplementary file 1 [file jcm-14-00468-s001.zip › jcm-3374200-supplementary.pdf]

Figures

Figure 1

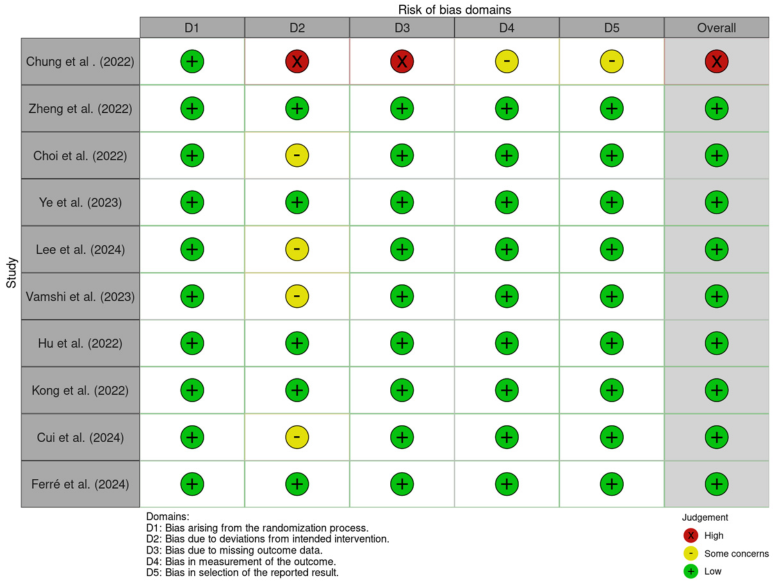

Fig. 1. Summary of the risk of bias assessment for included studies across five domains using the Cochrane Risk of Bias 2.0 (RoB 2) tool. Domains assessed include: D1 (bias arising from the randomization process), D2 (bias due to deviations from the intended intervention), D3 (bias due to missing outcome data), D4 (bias in measurement of the outcome), and D5 (bias in the selection of the reported result). Judgments are categorized as low risk (green), some concerns (yellow), and high risk (red). The overall risk of bias for each study is also reported in the rightmost column.

Figure 2

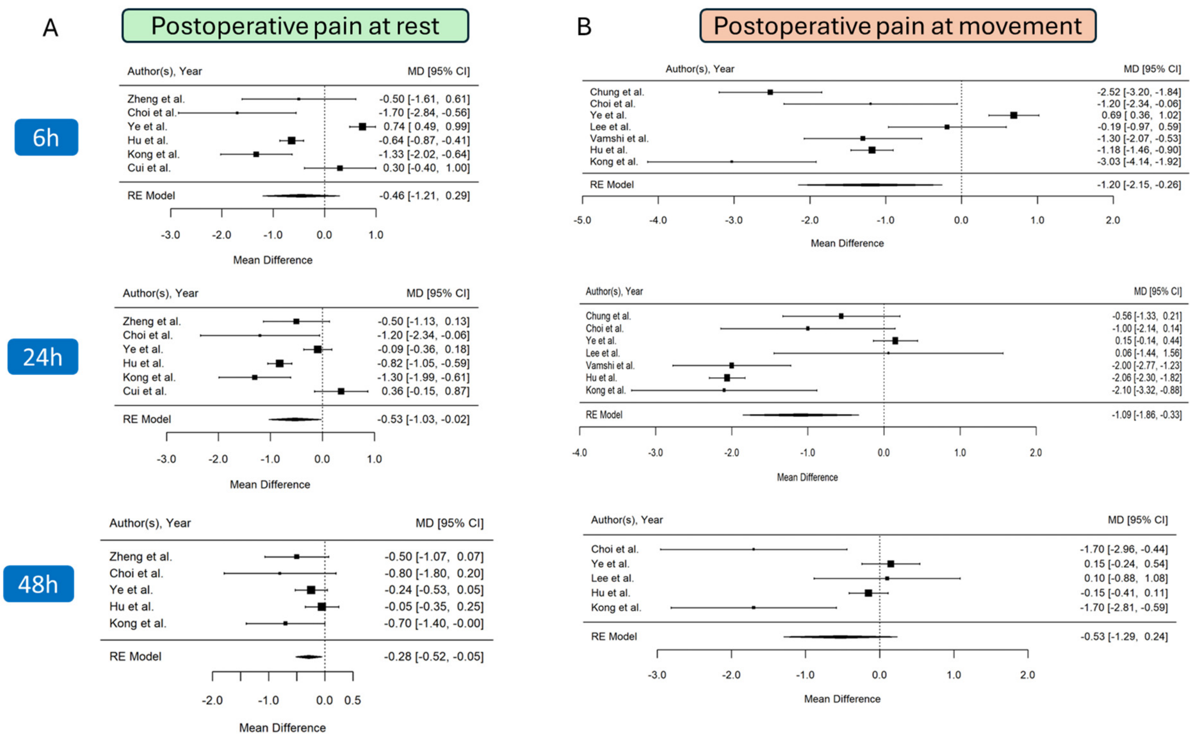

Fig. 2. Forest plot displaying pooled effect sizes for postoperative VAS scores at rest (A) and dynamic pain scores (B), measured at 6, 24, and 48 hours after surgery.

Figure 3

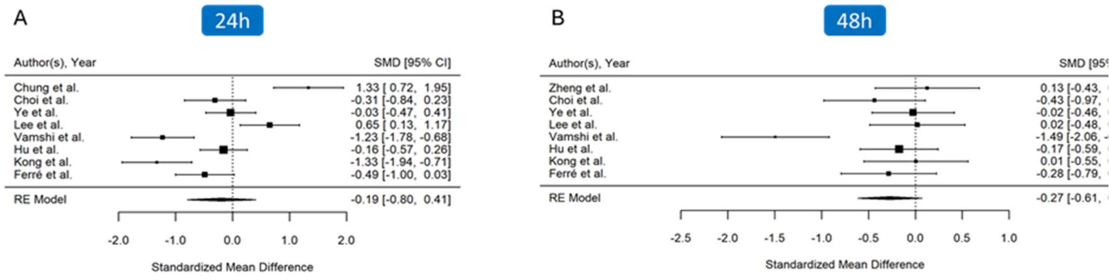

Fig. 3. Forest plot displaying pooled effect sizes for postoperative cumulative opioid consumption at 24 and 48 hours after surgery. A) At 24 hours post-surgery, the PENG block group showed a reduction in cumulative opioid consumption, though this reduction did not reach statistical significance. B) By 48 hours, the reduction in opioid consumption remained non-significant. A substantial heterogeneity was present among studies.

Figure 4

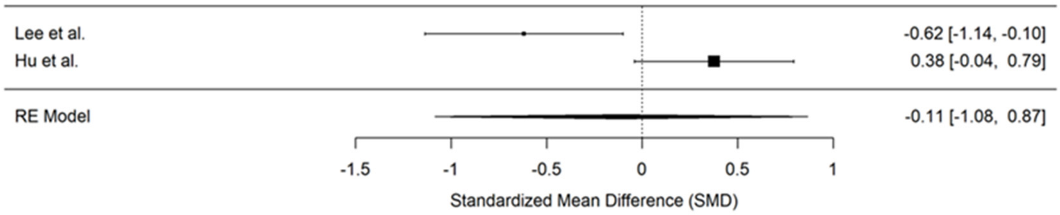

Fig. 4. Forest plot displaying pooled effect sizes for time to first analgesia request post-surgery.

Figure 5

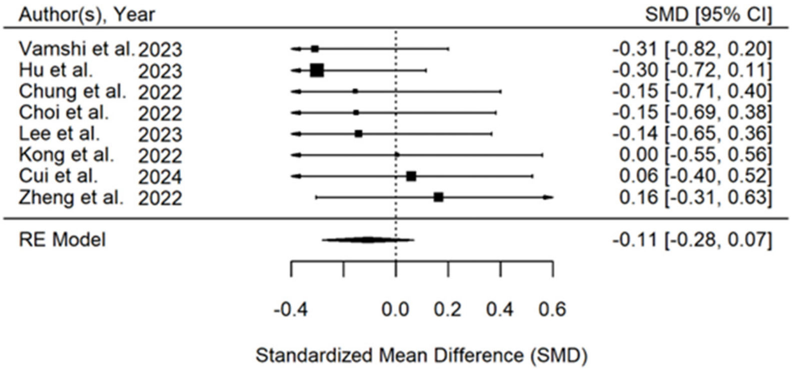

Fig. 5. Forest plot displaying pooled effect sizes for the duration of surgery in patients receiving PENG block versus control. The analysis included eight studies [24, 25, 27- 32] and showed no significant difference in the duration of surgery between the PENG block group and controls.

Figure 6

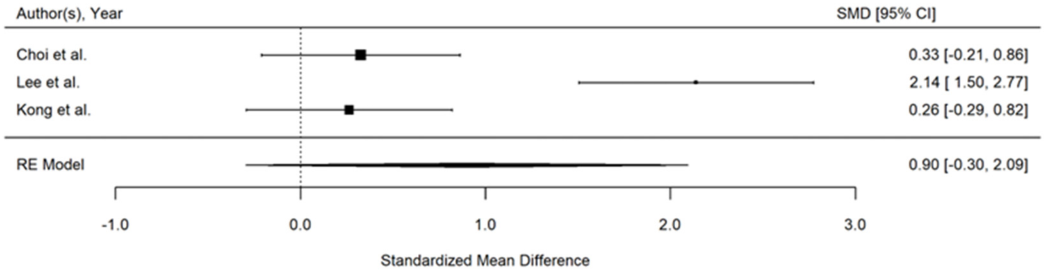

Fig. 6. Forest plot displaying the pooled effect sizes for patient satisfaction. The satisfaction data were standardised to a common scale (0 to 10) for comparison. Although there was an increase in satisfaction levels among patients who received the PENG block, this improvement did not reach statistical significance.

Figure 7

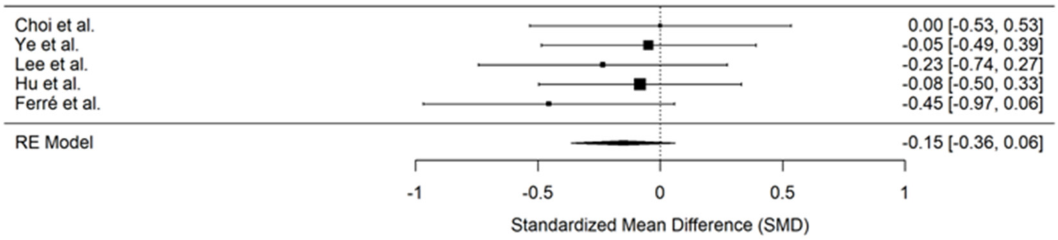

Fig.7 Forest plot displaying pooled effect sizes for the length of hospital stay (in hours). The PENG block group showed a slight reduction in the length of hospital stay compared to the control group, but this reduction was not statistically significant.

Figure 8

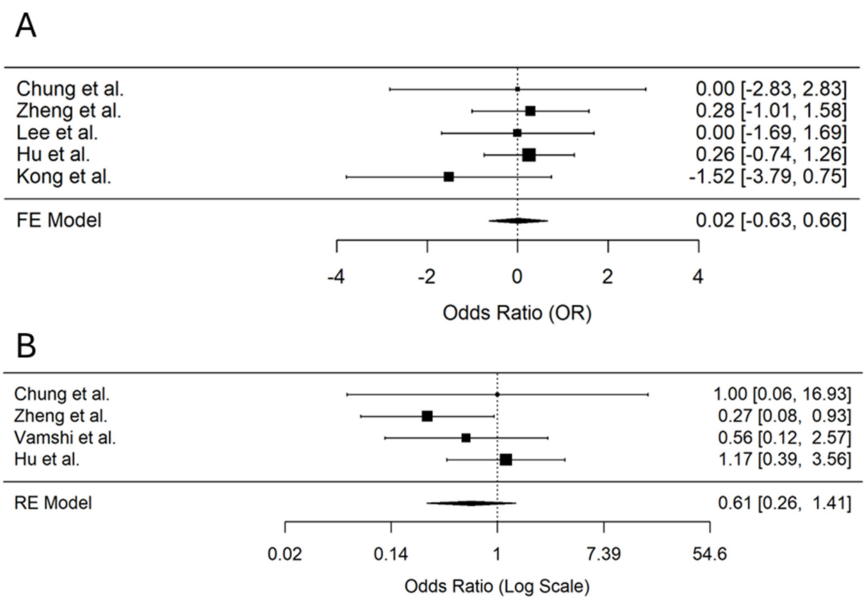

Fig. 8 Forest plots displaying the pooled odds ratios (OR) for postoperative nausea (A) and vomiting (B) in patients receiving PENG block versus control.

Supplementary Figure A1

Appendix A

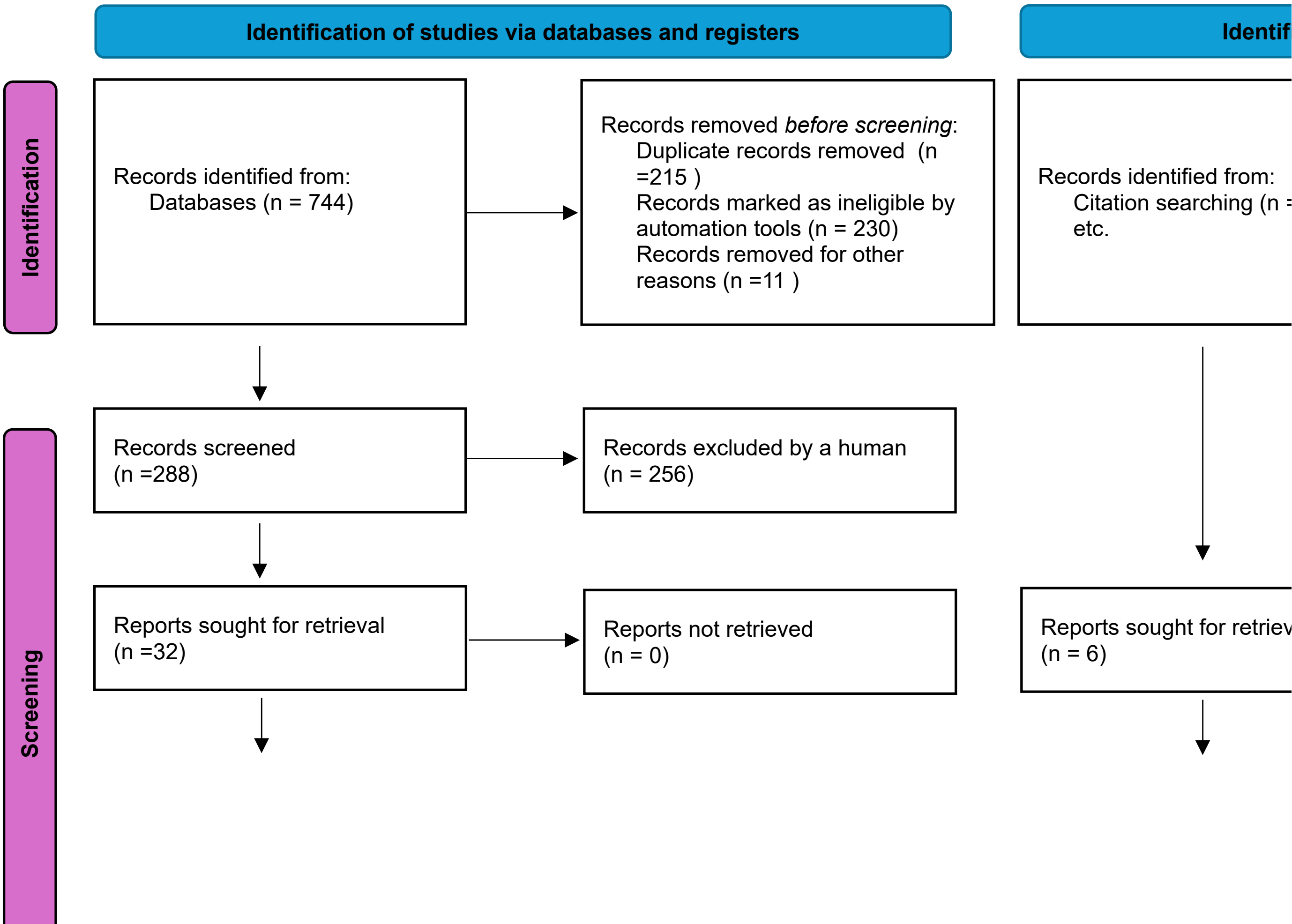

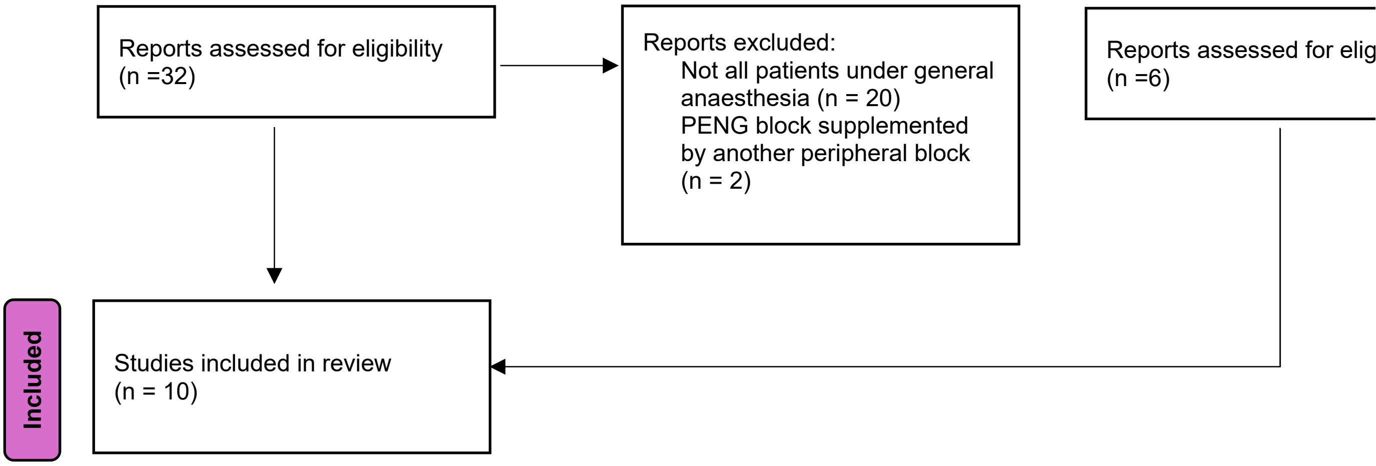

Supplementary Figure A1. **PRISMA 2020 flow diagram for new systematic reviews which included searches of databases, registers and other sources**

Supplementary Figure 1B

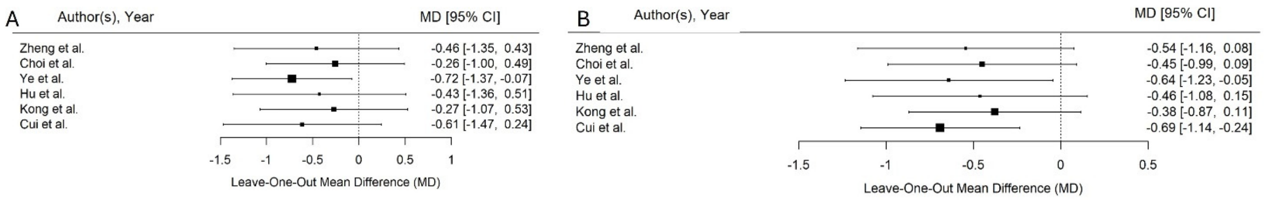

Supplementary Fig. B1. Sensitivity analysis of mean differences (MD) in pain scores during rest at 6 hours (A) and 24 hours (B) post-surgery.
